# Efficacy and safety of mesenchymal stromal cells in preclinical models of acute lung injury: a systematic review protocol

**DOI:** 10.1186/2046-4053-3-48

**Published:** 2014-05-23

**Authors:** Manoj M Lalu, David Moher, John Marshall, Dean Fergusson, Shirley HJ Mei, Malcolm Macleod, Gilly Griffin, Alexis F Turgeon, Michael Rudnicki, Jason Fishman, Marc T Avey, Becky Skidmore, Jeremy M Grimshaw, Duncan J Stewart, Kavita Singh, Lauralyn McIntyre

**Affiliations:** 1 Department of Anesthesiology, University of Ottawa, Ottawa, ON, Canada; 2 Department of Medicine (Division of Critical Care), University of Ottawa, Ottawa, ON, Canada; 3 Regenerative Medicine Program, The Ottawa Hospital Research Institute, Ottawa, ON, Canada; 4 Department of Cell and Molecular Medicine, University of Ottawa, Ottawa, ON, Canada; 5 Clinical Epidemiology Program, The Ottawa Hospital Research Institute, Ottawa, ON, Canada; 6 Department of Surgery (Critical Care), University of Toronto, Ottawa, ON, Canada; 7 Department of Epidemiology and Community Medicine, University of Ottawa, Ottawa, ON, Canada; 8 Division of Clinical Neurosciences, University of Edinburgh, Edinburgh, UK; 9 Department of Medicine, University of Ottawa, Ottawa, ON, Canada; 10 Canadian Council on Animal Care, Ottawa, ON, Canada; 11 Population Health and Optimal Health Practices Unit (Trauma – Emergency – Critical Care Medicine), Centre de Recherche du CHU de Québec (Enfant-JésusHospital), Université Laval, Québec, QC, Canada; 12 Division of Critical Care Medicine, Department of Anesthesiology, Université Laval, Québec, QC, Canada; 13 Department of Medicine (Critical Care), Ottawa Hospital, Ottawa Hospital Research Institute, Centre for Transfusion and Critical Care Research, Canadian Blood Services, 501 Smyth Road, Box 201, Ottawa, ON K1H 8 L6, Canada; 14 University of Ottawa, 451 Smyth Road, Ottawa, ON K1H 8 M5, Canada; 15 Independent consultant, Ottawa, ON, Canada

**Keywords:** Mesenchymal stromal cells, Mesenchymal stem cells, Acute lung injury, Acute respiratory distress syndrome, Preclinical, Systematic review protocol

## Abstract

**Background:**

Acute respiratory distress syndrome (ARDS) in humans is caused by an unchecked proinflammatory response that results in diffuse and severe lung injury, and it is associated with a mortality rate of 35 to 45%. Mesenchymal stromal cells (MSCs; ‘adult stem cells’) could represent a promising new therapy for this syndrome, since preclinical evidence suggests that MSCs may ameliorate lung injury. Prior to a human clinical trial, our aim is to conduct a systematic review to compare the efficacy and safety of MSC therapy versus controls in preclinical models of acute lung injury that mimic some aspects of the human ARDS.

**Methods/Design:**

We will include comparative preclinical studies (randomized and non-randomized) of acute lung injury in which MSCs were administered and outcomes compared to animals given a vehicle control. The primary outcome will be death. Secondary outcomes will include the four key features of preclinical acute lung injury as defined by the American Thoracic Society consensus conference (histologic evidence of lung injury, altered alveolar capillary barrier, lung inflammatory response, and physiological dysfunction) and pathogen clearance for acute lung injury models that are caused by infection. Electronic searches of MEDLINE, Embase, BIOSIS Previews, and Web of Science will be constructed and reviewed by the Peer Review of Electronic Search Strategies (PRESS) process. Search results will be screened independently and in duplicate. Data from eligible studies will be extracted, pooled, and analyzed using random effects models. Risk of bias will be assessed using the Cochrane risk of bias tool, and individual study reporting will be assessed according to the Animal Research: Reporting of *In Vivo* Experiments (ARRIVE) guidelines.

**Discussion:**

The results of this systematic review will comprehensively summarize the safety and efficacy of MSC therapy in preclinical models of acute lung injury. Our results will help translational scientists and clinical trialists to determine whether sufficient evidence exists to perform a human clinical trial. These results may also guide future acute lung injury preclinical and clinical research.

## Background

Acute respiratory distress syndrome (ARDS) is a devastating clinical condition caused by an acute, diffuse, and severe lung injury that requires management in the intensive care unit. It is characterized by unchecked inflammation that leads to severe hypoxemia, pulmonary alveolar and capillary membrane leakage, and progressive respiratory failure [1,2]. Approximately 200,000 new cases are identified in the United States each year [3] and it is a leading cause of death with mortality rates of approximately 35 to 45% [4].Significant long-term sequelae for survivors include physical, psychological, and emotional dysfunction that results in only half of previously healthy individuals returning to employment at one year after their illness [3,5,6]. The ARDS also carries a significant economic burden, as it has the highest cost per case of any acute care condition currently treated [7].

There are over 50 different causes of ARDS (see Table 1), with sepsis and pulmonary aspiration being the most common [8,9]. The pathophysiology of ARDS is complex, but its hallmarks include lung endothelial and alveolar epithelial injury with a consequent increase in membrane permeability and the accumulation of protein-rich debris in the alveolar air space [10]. Injury to the epithelium and endothelium is largely caused by a complex and exaggerated production of many inflammatory mediators (for exampleTNF-α, IL-6) [8,11,12]. These host processes are likely responsible for the high rates of morbidity and mortality of this illness.

**Table 1:** Examples of causes of acute lung injury in humans

**Direct**	**Indirect**
Infectious pneumonia	Sepsis
Pulmonary contusion	Severe trauma
Aspiration	Surface burns
Smoke inhalation	Venous air embolism
Near drowning	Amniotic fluid embolism
Following upper airway obstruction	Neurogenic pulmonary edema
Acute eosinophilic pneumonia	Multiple blood transfusions
Bronchiolitis obliterans organizing pneumonia (BOOP)	Leukoagglutinin reactions
Miliary tuberculosis	Pancreatitis
	Drug reaction/overdose
	Cardiopulmonary bypass
	Multiple fractures
	Postbone marrow transplantation

Supportive care strategies are the mainstay of therapy for ARDS. These include low tidal volume mechanical ventilation, restrictive fluid restriction strategies, and more recently prone positioning and paralysis [13-16]. Despite these interventions, the mortality for ARDS remains considerably high. Thus, the evaluation of novel therapeutics is required to reduce morbidity and death associated with this devastating syndrome [10]. Mesenchymal stromal cells (MSCs) may represent one novel therapy for this inflammatory condition.

MSCs (also known as ‘adult stem cells’, marrow stromal cells, or mesenchymal stem cells) have been well characterized and may be isolated from virtually every tissue type, including bone marrow, adipose tissue, and the umbilical cord [17]. *In vivo*,endogenous MSCs contribute to vascular homeostasis and respond to inflammation [18]. When cultured *ex vivo* and administered in larger numbers, MSCs are highly immunomodulatory [19]. In preclinical research, models described as ‘acute lung injury’ are used to study the clinical entity of ARDS. In these preclinical acute lung injury models, MSCs modulate the inflammatory response, augment tissue repair, enhance pathogen clearance, and reduce severity of injury, pulmonary dysfunction, and death [20-29].

As a prelude to a human clinical trial we will conduct a systematic synthesis of MSC therapy for preclinical acute lung injury. These data will help to determine whether there is a sufficient range of methodologically rigorous evidence to support a clinical trial administering MSCs in humans with ARDS. These data may also help to guide the objectives and designs of future preclinical and clinical research.

### Study question

In controlled preclinical studies of acute lung injury, do MSCs reduce death and the severity of acute lung injury as described by the American Thoracic Society preclinical acute lung injury consensus conference [30], and do they enhance pathogen clearance in infectious acute lung injury models?

## Methods and design

### Protocol and registration

The systematic review and meta-analysis protocol was developed through discussions with our scientific research team of clinical (LM, DF) and preclinical research scientists (ML,SM, DS), an information specialist (BS), experts in knowledge synthesis and translation (DM, JG, MM, KS, MA), knowledge users from the Canadian Critical Care Trials Group (CCCTG; JM), the Canadian Stem Cell Network (MR), and the Canadian Council on Animal Care (CCAC; GG), and the Canadian Critical Care Translational Biology Group(CCCTBG; http://www.ccctbg.ca). It is listed on the Collaborative Approach to MetaAnalysis and Review of Animal Data from Experimental Studies (CAMARADES)website (http://www.camarades.info).

### Study eligibility criteria

We will include controlled comparative studies (randomized, quasi-randomized, and non-randomized) of preclinical acute lung injury that evaluate the efficacy and safety outcomes of MSC treatment.

### Preclinical model eligibility criteria

We will include all preclinical *in vivo* animal models of experimentally induced acute lung injury that mimic at least some aspects of the pathophysiology of humans with ARDS according to the American Thoracic Society consensus criteria [30]. The inclusion of a wide range of acute lung injury animal models should enhance the generalizability of our study findings. Acute lung injury in animal models may be induced by several experimental methods (Table 2). These include direct(for example intratracheal bacteria or endotoxin), or indirect (for example intravenous or intraperitoneal bacteria or endotoxin) induction of infectious acute lung injury, and induction of injury by the ventilator (ventilator-induced lung injury), chemicals, or chemotherapeutic agents(for example bleomycin, oleic acid, hydrochloric acid),trauma, shock (for example hemorrhagic), or remote organ injury (for example pancreatitis, ischemia reperfusion). We will exclude neonatal animal models of acute lung injury since our proposed future clinical trial will focus on adults with ARDS and because the mechanisms of disease, and efficacy of treatments, are likely to be different in this group.

**Table 2:** Examples of animal models of acute lung injury

**Type of injury**	**Example of model**
Infection in lung	Intratracheal live bacteria
Bacterial components in lung	Intratracheal endotoxin
Systemic infection	Cecal ligation and puncture
Systemic bacterial components	Systemic endotoxin
Induction by ventilator	Ventilator-induced acute lung injury
Chemical or chemotherapeutic	Oleic acid
	Hydrochloric acid
	Bleomycin
Shock	Hemorrhagic
Trauma	Chest trauma
Remote organ injury	Ischemia reperfusion
	Pancreatitis

### Intervention

The preclinical intervention group will include animals from studies that examine MSC celltypes (xenogeneic, syngeneic, or allogeneic cells from any tissue source). MSCs will be defined using minimal criteria set out in the International Society for Cellular Therapy (ISCT) consensus statement [31]. MSCs must be administered during or following the induction of experimental acute lung injury. Experiments using pretreatment of MSCs will be excluded since they are clinically relevant for the prevention, but not the treatment, of human ARDS. In order to focus on non-manipulated MSCs, studies using either differentiated MSCs (for example MSCs that have been differentiated to an endothelial cell) or MSCs engineered to over- or under-express particular genes, or studies using a co-treatment with another therapy or cell type will be excluded.

### Comparison

The preclinical comparison group will include animals from studies that have had experimental acute lung injury induced but have not been administered MSCs. This will allow us to perform effect size calculations in our meta-analysis to examine how efficacious and safe these cells are in the acute lung injury. We will use other control groups, such as healthy animals or sham-injured controls, to examine the severity of preclinical acute lung injury.

### Preclinical primary endpoint: death

The primary endpoint is death, measured at specific time points after administration of the MSCs or control intervention. Time of death will be categorized as less than 2 days, between 2 to 4 days, and greater than 4 days. We will also measure all deaths that occur by the end of the follow-up period to give a measure of overall mortality. Death is a clinically meaningful endpoint given the high mortality rate of clinical ARDS [3]. The timing of our mortality assessments is intended to capture the burden of both early and late deaths as is typically seen in clinical trials of humans with acute lung injury (for example intensive care unit mortality, 28-day mortality, and 90-day mortality). The timing for these death assessments also reflects when the majority of deaths occur in the acute lung injury experimental animal setting. For example, one preclinical study demonstrated that mortality at 48 hours was 60%, and the majority of deaths occurred between 30 to 40 hours post-experimental induction of acute lung injury [29].

### Preclinical secondary endpoints

A consensus statement by the American Thoracic Society describes four key features that define acute lung injury in the animal model along with specific measurements to assess for the presence of each feature [30]. These are summarized in Table 3. Our secondary endpoints will include the four main features and focus on the ‘very relevant’ measurements defined by the consensus criteria [30]. These include histologic evidence of lung injury (for example lung injury score), alteration of the alveolar capillary barrier (for example increased concentration of high molecular weight proteins in bronchoalveolar fluid),measures of the inflammatory response in the lung (for example pulmonary neutrophils, cytokines), and measures of physiological dysfunction (for example alveolar-arterial gradient of oxygen concentration). The American Thoracic Society consensus statement does not specify which lung inflammatory cytokines to measure. Hence, our team of translational cell and acute lung injury scientists (JM, SM, DS,ML) established consensus on what cytokines would be considered ‘most relevant’ for analysis in our systematic review (see Table 3).

**Table 3:** Features and measurements of acute lung injury in animal models

**Feature**	**‘Very relevant’ measurements**
Histological evidence of tissue injury	Accumulation of neutrophils in the alveolar or the interstitial space
	Formation of hyaline membranes
	Presence of proteinaceous debris in the alveolar space (such as fibrin strands)
	Thickening of the alveolar wall
	Enhanced injury as measured by a standardized histology score
Alteration of the alveolar capillary barrier	An increase in extravascular lung water content
	Accumulation of an exogenous protein or tracer in the airspaces or the extravascular compartment
	Increase in total bronchoalveolar lavage (BAL) protein concentration
	Increase in concentration of high molecular weight proteins in BAL fluid (for example albumin, IgM)
	Increase in the microvascular filtration coefficient
Inflammatory response	Increase in the absolute number of neutrophils in BAL fluid
	Increase in lung myeloperoxidase (MPO) activity or protein concentration
	Increase in the concentrations of cytokines in lung tissue or BAL fluid (IFN-γ, TNF-α, IL-6, IL-1β, chemokine (C-X-C motif) ligand2, chemokine (C-C motif) ligand 2, IL-8, IL-10, prostaglandin E2, IL-1 receptor antagonist)
Physiological dysfunction	Hypoxemia
	Increased alveolar-oxygen difference

Modified from the American Thoracic Society consensus statement [30]. This workshop was held with translational investigators to identify the defining features and measurements for acute lung injury animal models to simulate the pathophysiology of human ARDS. ARDS, acute respiratory distress syndrome; BAL, bronchoalveolar lavage; IFN, interferon; IgM, immunoglobulin M; IL, interleukin; MPO, myeloperoxidase; TNF, tumor necrosis factor.

For animal models that use infectious induction of acute lung injury, we will describe pathogen clearance (for example number of bacterial colony forming units) in different tissue beds, since animal sepsis models suggest that this is increased by MSCs [32-34].

All secondary endpoints will be analyzed in categories of time from the induction of acute lung injury of less than 6 hours, between 6 and 24 hours, between greater than 24 and 72 hours, and day 7 after the administration of MSCs versus controls. These time points for measurement were selected since the development of inflammation and acute lung injury occurs over time and contributes to death and morbidity in this population [2]. Where reported, the occurrence of adverse events will also be documented for each included study.

### Information sources

Search strategies will be developed and tested through an iterative process by an experienced medical information specialist in consultation with the review team. Using the Ovid platform, we will search Ovid MEDLINE®, Ovid MEDLINE® In-Process & Other Non-Indexed Citations, and Embase Classic plus Embase. We will also search BIOSIS and Web of Science using Web of Knowledge. The strategy will be reviewed by another senior information specialist using the Peer Review of Electronic Search Strategies (PRESS) template [35].

Search strategies will use a combination of controlled vocabulary (for examplemesenchymal stem cells, acute lung injury, respiratory distress syndrome) and keywords (for example MSCs, ALI, acute/adult respiratory distress syndrome). Vocabulary and syntax will be adjusted across the databases. Two recently published animal filters [36,37], validated for PubMed/MEDLINE and Embase and amended slightly, will be used to increase relevancy. These filters will be adjusted for use in the other databases where a validated filter is unavailable. There will be no language or date restrictions on any of the searches. We will perform a grey literature search of selected conference websites not covered in the aforementioned databases, and will search the websites of animal research organizations. We will also search Mendeley and Google Scholar. The proposed Ovid search appears in Additional file 1.

The research team will contact authors of included studies to invite the further contribution of any unpublished data relevant to this review. The bibliographies of included studies and pertinent reviews will also be hand searched for further preclinical studies. Unpublished studies will be described in the results section but data from these studies will be included in any quantitative analyses. The research team will also contact biotechnology companies (for example Osiris Therapeutics, Columbia, MD, USA) that produce MSCs to identify further unpublished studies or studies that are currently ongoing.

### Study selection

The titles and abstracts of search results will be screened independently by two investigators. The fulltext of all potentially eligible studies will be retrieved and reviewed for eligibility, independently, by two members of the team using the *a priori* inclusion criteria described above. Disagreements between reviewers will be resolved by consensus or by a third member of the systematic review team (LM or ML). Reasons for exclusion of potentially eligible studies will be recorded to enable a transparent selection process and to be in accordance with the Preferred Reporting Items for Systematic Reviews and Meta-Analyses (PRISMA) guidelines developed for proper reporting of clinical systematic reviews [38].

### Data collection and process and data items

Data will be extracted independently by two members of the research team into standardized, pilot-tested forms. Specific data elements are listed in Table 4.

**Table 4:** Data collection elements

**Category**	**Specific items**
Study characteristics	Study title, author, date of publication, journal published, sponsorship, country of publication
Study population (animal model)	Animal type, age, gender, strain, and weight, presence of co-morbid illnesses
Type of acute lung injury model	Direct infection, indirect infection, ventilator-induced injury, chemical-induced injury, trauma, shock, pancreatitis, ischemia-reperfusion
Severity of experimentally induced acute lung injury	According to the lung injury score [16]
Intervention and comparison	Time and route given, description of preparation and suspension of MSCs and controls
Co-interventions	Resuscitation fluids, antibiotics, and mechanical ventilation
Preclinical endpoints	Death, features and measures of acute lung injury (Table 3) that include: histological evidence of pulmonary injury;alteration in alveolar capillary barrier;pulmonary and systemic inflammatory response; measurements of physiological dysfunction; and pathogen clearance (measured using the number of bacterial colony forming units in lung, liver, spleen, and blood), and adverse events
Risk of bias assessments	In accordance with the Cochrane risk of bias tool, allocation concealment, randomization, blinding (personnel, endpoint measurements), and endpoint measures (completeness of follow-up)
Quality of reporting of individual preclinical studies	In accordance with elements of the ARRIVE guidelines [42]
Other	Industry sponsorship, single centre versus multi-centre, and presence of *a priori* sample size calculation.

ARRIVE, Animal Research: Reporting of *In Vivo* Experiments; MSC, mesenchymal stromal cell.

### Assessment of risk of bias

Risk of bias will be evaluated independently by two reviewers for each included preclinical study. Since no validated and standardized risk of bias checklist exists for preclinical studies, we will describe the biases of the included studies using the Cochrane risk of bias assessment tool [39]. Items in this tool include assessments for concealment of allocation, random sequence generation, blinding of personnel and the endpoint measurements, and completeness of endpoint reporting. Each bias criterion will be assigned a value of low, high, or unclear risk of bias for each included study [39].

### Assessment of construct validity and external validity

We will also record features that will facilitate judgements of construct validity and external validity [40]. Construct validity in preclinical research concerns the extent to which an experimental system accurately models a clinical entity. These will include: type, age, gender, and strain of animal; presence of co-morbidities; type of acute lung injury model; timing, dose and mode of MSC administration; and use of co-interventions (for example fluid resuscitation, use of antibiotics for infectious acute lung injury models) (Table 5). External validity in preclinical research concerns the extent to which cause and effect relationships holdup under varied conditions [41]. In our study this will include the use of a multi-centre preclinical study (Table 5).

**Table 5:** Elements of construct validity and external validity

**Category**	**Specific items**
Death	True death versus surrogate endpoints
Animal species	Mouse, rat, sheep, pig, other
Strain	Example for mice: BALB/c versus C57Bl/6
Animal age	Mature adult versus middle-aged versus older adult for each species (for example mouse 3 to 10 months, 10 to 18 months, 18 to 24 months)
Gender	Male versus female versus mix of genders used
Model of acute lung injury	See Table 2
Presence of co-morbidities	Yes versus no
Severity of lung injury	Lung injury score
MSC preparation	Fresh versus fresh from previously cryopreserved versus thawed cryopreserved product
Timing of MSC administration following induction of acute lung injury	0 to 1 hours versus 1 to 6 hours versus >6 hours
Route of MSC administration	Intravenous versus intratracheal versus intraperitoneal versus intramuscular
Type of control	PBS versus normal saline versus fibroblasts versus heat-killed MSCs
Use of co-interventions	Resuscitation fluids: yes versus no Use of antibiotics in infectious models: yes versus no
Mechanical ventilation	Yes versus no
Number of participating study centers	Single versus multi-center

MSC, mesenchymal stromal cell; PBS, phosphate buffered saline.

### Description of reporting

We will describe the quality of reporting of the included preclinical studies using the elements of the Animal Research: Reporting of *In Vivo* Experiments (ARRIVE) guidelines. The ARRIVE guidelines were developed to enhance the transparent and comprehensive reporting of research methods and results for *in vivo* animal experiments [42].

### Data analysis

Where appropriate, dichotomous endpoints (for example death) from each included study will be pooled and described as odds ratios and 95% confidence intervals incorporating a random effects modeling approach with the use of forest plots for presentation of the data [43]. Continuous endpoints will be pooled using the ratio of weighted means method with inverse variance random effects modeling [44]. Statistical heterogeneity of included preclinical studies will be measured using the I^2^ test with 95% uncertainty intervals [45]. If there are sufficient number of studies (≥10), an evaluation for the presence of publication bias will be conducted with funnel plot techniques, and Egger’s regression test [46].

Sensitivity analyses to examine heterogeneity on the primary endpoint death will be carried out according to risk of bias assessments. Several subgroup analyses to examine preclinical heterogeneity will be conducted on the primary endpoint death (see Table 5). Where appropriate, analyses will include: definition of death; the type of animal model; animal age [47], gender, and strain; presence of co morbidities; type of experimental induction of acute lung injury (Table 2); severity of the acute lung injury model [30]; MSC preparation; timing of administration of MSCs from induction of acute lung injury; route of MSC administration; type of controls; use of co-interventions, antibiotics, and mechanical ventilation; single versus multi-centre study; and presence of an *a priori* sample size calculation. These subgroup analyses are exploratory and the results will be interpreted with caution.

### Knowledge translation

The results of this systematic review will be of interest to a broad audience, and we have identified several knowledge users. We have planned an end of study knowledge translation workshop where our key findings will be disseminated to the group members and key stakeholders. In addition, this workshop will identify further avenues of dissemination and determine future research directions.

Our Principal Knowledge user (JM) will help facilitate presentation of study results at national and international critical care meetings. Two other knowledge users will aid the international dissemination of results through the Canadian Stem Cell Network, internationally through CellCAN (MR), and through the International Council for Laboratory Animal Science (ICLAS; GG) which encourages better reporting and translation of animal science.

## Discussion

The results of this systematic review will inform translational and clinical scientists, clinicians, and health regulators internationally regarding the existing preclinical evidence for MSC therapy in acute lung injury. Our review is timely since there is an increasing amount of research dedicated to the evaluation or MSCs in many clinical domains [48]. Many therapeutics that appear promising in preclinical studies fail to translate into successful therapies. In a systematic review of highly cited animal studies, only one-third of studies translated to human randomized controlled trials (RCTs) [49]. This lack of translation may, in part, be related to failure to acknowledge the limitations of preclinical studies [50], as well as methodological flaws that bias the treatment effect in clinical trials that may also apply to preclinical studies. For example, preclinical interventional research in stroke and emergency medicine suggeststhat these weaknesses are associated with overestimates of the effect size for different treatments and publication bias [50,51]. Hence, our review is critical before spending the substantial energy and funding that is required to conduct a clinical trial. In a broader perspective, we also hope this review will identify challenges and barriers related to the conduct of these preclinical studies. Ultimately, our team will inform and enrich future preclinical and clinical MSC research that should aid the translation of this novel therapeutic in clinical trials.

## Abbreviations

ARDS: Acute respiratory distress syndrome; ARRIVE: Animal Research: Reporting of *In Vivo* Experiments; BAL: Bronchoalveolar lavage; BOOP: Bronchiolitis obliterans organizing pneumonia; CAMARADES: Collaborative Approach to Meta Analysis and Review of Animal Data from Experimental Studies; CCAC: Canadian Council on Animal Care; CCCTBG: Canadian Critical Care Translational Biology Group; CCCTG: Canadian Critical Care Trials Group; ICLAS: International Council for Laboratory Animal Science; IFN: Interferon; IgM: Immunoglobulin M; IL: Interleukin; ISCT: International Society for Cellular Therapy; MPO: Myeloperoxidase; MSC: Mesenchymal stromal cell; PBS: Phosphate buffered saline; PRESS: Peer Review of Electronic Search Strategies; PRISMA: Preferred Reporting Items for Systematic Reviews and Meta-Analyses; RCT: Randomized controlled trial; TNF: Tumor necrosis factor.

## Competing interests

DS is President and CEO of Northern Therapeutics (Montréal, QC, Canada). SM is an employee of Northern Therapeutics. MM is a member of the UK Home Office Animals in Science Committee. The remaining authors have no competing interests to declare.

## Authors’ contributions

LM, ML, and DM conceived the study design. ML and LM were responsible for initial drafting and manuscript revisions. ML, JF, and LM were responsible for the data collection. JMG, AFT, KS, BS, MA, JM, GG, and DF provided critical revisions and statistical support. MM provided expertise in the design of preclinical systematic reviews. JM, DS, SM, and MR provided translational biology expertise, and provided oversight for planned eligibility criteria and outcome measures. JM, MR, and GG will oversee knowledge translation. All authors reviewed several drafts of the manuscript and approved the final version.

## Supplementary Material

Additional file 1 **Representative search strategy.** Databases: Embase Classicplus Embase, 1947 to 5 June 2013; and Ovid MEDLINE In-Process & Other Non-Indexed Citations and Ovid MEDLINE, 1946 to present. Click here for file
