# Supplementary material for: Efficacy and safety of mesenchymal stromal cells in preclinical models of acute lung injury: a systematic review protocol
Source: Syst Rev. 2014 May 23;3:48. doi: 10.1186/2046-4053-3-48 (PMC4046388; doi:10.1186/2046-4053-3-48)
Supplement: Additional file 1 — Representative search strategy. Databases: Embase Classicplus Embase, 1947 to 5 June 2013; and Ovid MEDLINE In-Process & Other Non-Indexed Citations and Ovid MEDLINE, 1946 to present. [file 2046-4053-3-48-S1.docx]

**Additional file 1 – Representative Search Strategy**

Database: Embase Classic+Embase <1947 to 2013 June 05>, Ovid MEDLINE(R) In-Process & Other Non-Indexed Citations and Ovid MEDLINE(R) <1946 to Present> Search Strategy:

--------------------------------------------------------------------------------

1 Mesenchymal Stem Cells/ (34040)

2 Mesenchymal Stem Cell Transplantation/ (8987)

3 Multipotent Stem Cells/ (5907)

4 Mesenchymal Stromal Cells/ (14974)

5 ((mesenchymal adj3 (stem or stroma$1 or progenitor*)) and (cell$1 or cell-induced)).tw. (44453)

6 (MSC or MSCs or ADMSC or ADMSCs or BM-MSC or BM-MSCs or BMD-MSC or BMD-MSCs or BMDMSC or BMDMSCs or BMSC or BMSCs or HBMSC or HBMSCs or ABMSC or ABMSCs or MAPC or MAPCs).tw. (35768)

7 (((multipotent or multi-potent) adj3 (stem or stroma$1 or progenitor*)) and (cell$1 or cell-induced)).tw. (8725)

8 ((marrow* adj3 (stem or stroma$1 or progenitor*)) and (cell$1 or cell-induced)).tw. (43339)

9 (exp Bone Marrow Cells/ or Bone Marrow Transplantation/) and (exp Stem Cells/ or exp Stem Cell Transplantation/ or Stem Cell Niche/) (77540)

10 (colony-forming unit fibroblast* or CFU-F$1).tw. (1415)

11 exp Mesoderm/cy [Cytology] (5943)

12 exp Adult Stem Cells/ (7455)

13 ((adult* adj3 (stem or stroma$1 or progenitor*)) and (cell$1 or cell-induced)).tw. (17001)

14 ((fibroblast-derived adj3 (stem or stroma$1 or progenitor*)) and (cell$1 or cell-induced)).tw. (40)

15 IPS cell$1.tw. (3176)

16 or/1-15 (174293)

17 exp Stem Cell Transplantation/ (128846)

18 exp Gene Therapy/ (96802)

19 Mesenchymal.tw. (116010)

20 (17 or 18) and 19 (11653)

21 16 or 20 (174617)

22 exp Lung/pa, pp [Pathology, Physiopathology] (73140)

23 exp lung diseases/ (1655002)

24 ((lung$1 or pulmonary) adj3 (disease* or injur* or contusion* or embolism* or haemorrhag* or hemorrhag* or infection* or trauma*)).tw. (338023)

25 exp Respiratory Distress Syndrome, Adult/ (34780)

26 ((acute or adult) adj respiratory distress syndrome).tw. (24402)

27 (ARDS or ARDSS).tw. (17412)

28 (shock adj1 lung).tw. (1093)

29 exp Acute Lung Injury/ (9987)

30 (lung injur* adj1 acute).tw. (19950)

31 ali.tw. (9000)

32 exp Respiratory Insufficiency/ (113996)

33 ((respiratory or ventilatory) adj3 (insufficien* or deficien* or disturbance* or dysfunction* or depression or failure or paralys#s)).tw. (97062)

34 exp Systemic Inflammatory Response Syndrome/ (261293)

35 ("inflammatory response syndrome" adj1 systemic).tw. (6469)

36 (sepsis or septic* or bacteremia or bacteraemia or endotoxic* or endotoxemi* or endotoxaemi*).tw. (293213)

37 exp Hyperoxia/ (9036)

38 hyperoxi*.tw. (16180)

39 exp Reperfusion Injury/ (64760)

40 ((pulmonary or lung$1 or nonpulmonary or non-pulmonary) adj3 (ischemi* or ischaemi* or reperfusion*)).tw. (4926)

41 exp Peritonitis/ (67720)

42 peritonitis.tw. (57018)

43 exp Pneumonia, Aspiration/ (14576)

44 ((pneumonia or acid) adj1 aspiration).tw. (7192)

45 (mendelson$1 adj syndrome).tw. (485)

46 exp Oleic Acid/ (24465)

47 (oleic acid* or oleate or 9-octadecenoic acid* or cis-9-octadecenoic acid*).tw. (35809)

48 exp Bleomycin/ (53023)

49 (bleomycin* or blanoxan or blenoxane or BLEO-cell or bleolem or bleomicin*).tw. (28063)

50 exp Endotoxins/ (117578)

51 (endotoxin* or ETX).tw. (72974)

52 exp Lipopolysaccharides/ (140803)

53 (lipopolysaccharide* or lipo-polysaccharide* or LPS or lipoglycan*).tw. (173627)

54 exp Bronchoalveolar Lavage Fluid/ (56822)

55 ((bronchoalveolar or broncho-alveolar or alveolar or bronchial or lung$1 or pulmonary or saline) adj1 lavage*).tw. (58750)

56 ((intravenous or IV or intrapulmonary or intra-pulmonary or intrabronchial or intra-bronchial) adj1 bacter*).tw. (592)

57 exp Cecum/ (35563)

58 exp Ligation/ or exp Punctures/ or in.fs. (378628)

59 ((cecum or cecal or coecum or caecum) adj3 (ligation* or puncture* or injur*)).tw. (5591)

60 (57 and 58) or 59 (6113)

61 exp Pancreatitis/ (118884)

62 Pancreatit*.tw. (104949)

63 (pancreas* adj3 inflam*).tw. (968)

64 or/22-56,60-63 (2787405)

65 21 and 64 (7053)

66 exp animal experimentation/ or exp models, animal/ or animals/ or mammals/ or vertebrates/ or exp fishes/ or exp amphibia/ or exp reptiles/ or exp birds/ or exp hyraxes/ or exp marsupialia/ or exp monotremata/ or exp scandentia/ or exp chiroptera/ or exp carnivora/ or exp cetacea/ or exp Xenarthra/ or exp elephants/ or exp insectivora/ or exp lagomorpha/ or exp rodentia/ or exp sirenia/ or exp Perissodactyla/ or primates/ or exp strepsirhini/ or haplorhini/ or exp tarsii/ or exp platyrrhini/ or catarrhini/ or exp cercopithecidae/ or gorilla gorilla/ or pan paniscus/ or pan troglodytes/ or exp pongo/ or exp hylobatidae/ or hominidae/ (10693999)

67 (animal$1 or chordata or vertebrate* or fish$2 or amphibian* or amphibium* or reptile$1 or bird$1 or mammal* or dog or dogs or canine$1 or cat or cats or hyrax* or marsupial* or monotrem* or scandentia or bat or bats or carnivor* or cetacea or edentata* or elephant* or insect or insects or insectivore or lagomorph* or rodent$2 or mouse or mice or murine or murinae or muridae or rat or rats or pig or pigs or piglet$1 or swine or rabbit$1 or sheep$1 or goat$1 or horse$1 or equus or cow or cows or cattle or calf or calves or bovine or sirenia or ungulate$1 or primate$1 or prosimian* or haplorhini* or tarsiiform* or simian*or platyrrhini or catarrhini or cercopithecidae or ape or apes or hylobatidae or hominid* or chimpanzee* or gorilla* or orangutan* or monkey or monkeys or ape or apes).tw. (8398088)

68 exp Drug Evaluation, Preclinical/ (307089)

69 (preclinic* or pre-clinic*).tw. (125267)

70 or/66-69 (12371409)

71 65 and 70 (4094)

72 71 use prmz (2127)

73 exp mesenchymal stem cell/ (34040)

74 exp mesenchymal stem cell transplantation/ (8987)

75 exp multipotent stem cell/ (19308)

76 exp mesenchymal stroma cell/ (1120)

77 ((mesenchymal adj3 (stem or stroma$1 or progenitor*)) and (cell$1 or cell-induced)).tw. (44453)

78 (MSC or MSCs or ADMSC or ADMSCs or BM-MSC or BM-MSCs or BMD-MSC or BMD-MSCs or BMDMSC or BMDMSCs or BMSC or BMSCs or HBMSC or HBMSCs or ABMSC or ABMSCs or MAPC or MAPCs).tw. (35768)

79 (((multipotent or multi-potent) adj3 (stem or stroma$1 or progenitor*)) and (cell$1 or cell-induced)).tw. (8725)

80 ((marrow* adj3 (stem or stroma$1 or progenitor*)) and (cell$1 or cell-induced)).tw. (43339)

81 (exp bone marrow cell/ or exp bone marrow transplantation/) and (exp stem cell/ or exp stem cell transplantation/) (80630)

82 (colony-forming unit fibroblast* or CFU-F$1).tw. (1415)

83 adult stem cell/ (5119)

84 ((adult* adj3 (stem or stroma$1 or progenitor*)) and (cell$1 or cell-induced)).tw. (17001)

85 ((fibroblast-derived adj3 (stem or stroma$1 or progenitor*)) and (cell$1 or cell-induced)).tw. (40)

86 IPS cell$1.tw. (3176)

87 exp mesoderm/ (29049)

88 (cell or cytolog*).tw. (5284561)

89 87 and 88 (13805)

90 or/73-86,89 (182688)

91 exp stem cell transplantation/ (128846)

92 exp gene therapy/ (96802)

93 Mesenchymal.tw. (116010)

94 (91 or 92) and 93 (11653)

95 90 or 94 (182999)

96 exp lung/ (494458)

97 (patholog* or physiopatholog* or physio-patholog*).tw. (1276913)

98 96 and 97 (30655)

99 exp lung disease/ (1655002)

100 ((lung$1 or pulmonary) adj3 (disease* or injur* or contusion* or embolism* or haemorrhag* or hemorrhag* or infection* or trauma*)).tw. (338023)

101 exp adult respiratory distress syndrome/ (34780)

102 ((acute or adult) adj respiratory distress syndrome).tw. (24402)

103 (ARDS or ARDSS).tw. (17412)

104 (shock adj1 lung).tw. (1093)

105 exp acute lung injury/ (9987)

106 (lung injur* adj1 acute).tw. (19950)

107 ali.tw. (9000)

108 exp respiratory failure/ (113996)

109 ((respiratory or ventilatory) adj3 (insufficien* or deficien* or disturbance* or dysfunction* or depression or failure or paralys#s)).tw. (97062)

110 exp systemic inflammatory response syndrome/ (261293)

111 ("inflammatory response syndrome" adj1 systemic).tw. (6469)

112 (sepsis or septic* or bacteremia or bacteraemia or endotoxic* or endotoxemi* or endotoxaemi*).tw. (293213)

113 exp hyperoxia/ (9036)

114 hyperoxi*.tw. (16180)

115 exp reperfusion injury/ (64760)

116 ((pulmonary or lung$1 or nonpulmonary or non-pulmonary) adj3 (ischemi* or ischaemi* or reperfusion*)).tw. (4926)

117 exp peritonitis/ (67720)

118 peritonitis.tw. (57018)

119 exp aspiration pneumonia/ (14576)

120 ((pneumonia or acid) adj1 aspiration).tw. (7192)

121 (mendelson$1 adj syndrome).tw. (485)

122 exp oleic acid/ (24465)

123 (oleic acid* or oleate or 9-octadecenoic acid* or cis-9-octadecenoic acid*).tw. (35809)

124 exp bleomycin/ (53023)

125 (bleomycin* or blanoxan or blenoxane or BLEO-cell or bleolem or bleomicin*).tw. (28063)

126 exp endotoxin/ (117578)

127 (endotoxin* or ETX).tw. (72974)

128 exp lipopolysaccharide/ (77890)

129 (lipopolysaccharide* or lipo-polysaccharide* or LPS or lipoglycan*).tw. (173627)

130 exp lung lavage/ (58846)

131 ((bronchoalveolar or broncho-alveolar or alveolar or bronchial or lung$1 or pulmonary or saline) adj1 lavage*).tw. (58750)

132 ((intravenous or IV or intrapulmonary or intra-pulmonary or intrabronchial or intra-bronchial) adj1 bacter*).tw. (592)

133 exp cecum/ (35563)

134 exp ligation/ (65710)

135 exp puncture/ (113102)

136 133 and (134 or 135) (1215)

137 ((cecum or cecal or coecum or caecum) adj3 (ligation* or puncture* or injur*)).tw. (5591)

138 exp Pancreatitis/ (118884)

139 Pancreatit*.tw. (104949)

140 (pancreas* adj3 inflam*).tw. (968)

141 or/98-132,136-140 (2778291)

142 95 and 141 (7609)

143 exp animal experiment/ or exp animal model/ or animal/ or exp invertebrate Chordata/ or exp experimental animal/ or exp transgenic animal/ or exp male animal/ or exp female animal/ or exp juvenile animal/ or vertebrate/ or exp fish/ or exp amphibia/ or exp reptile/ or exp bird/ or mammal/ or exp hyrax/ or exp marsupial/ or exp monotremate/ or exp scandentia/ or placental mammals/ or exp bat/ or exp carnivora/ or exp cetacea/ or exp edentata/ or exp elephant/ or exp insectivora/ or exp lagomorph/ or exp rodent/ or exp sirenia/ or exp ungulate/ or primate/ or exp prosimian/ or haplorhini/ or exp tarsiiform/ or simian/ or exp platyrrhini/ or catarrhini/ or exp cercopithecidae/ or ape/ or exp hylobatidae/ or hominid/ or exp chimpanzee/ or exp gorilla/ or exp orang utan/ (10715704)

144 (animal$1 or chordata or vertebrate* or fish$2 or amphibian* or amphibium* or reptile$1 or bird$1 or mammal* or dog or dogs or canine$1 or cat or cats or hyrax* or marsupial* or monotrem* or scandentia or bat or bats or carnivor* or cetacea or edentata* or elephant* or insect or insects or insectivore or lagomorph* or rodent$2 or mouse or mice or murine or murinae or muridae or rat or rats or pig or pigs or piglet$1 or swine or rabbit$1 or sheep$1 or goat$1 or horse$1 or equus or cow or cows or cattle or calf or calves or bovine or sirenia or ungulate$1 or primate$1 or prosimian* or haplorhini* or tarsiiform* or simian*or platyrrhini or catarrhini or cercopithecidae or ape or apes or hylobatidae or hominid* or chimpanzee* or gorilla* or orangutan* or monkey or monkeys or ape or apes).tw. (8398088)

145 (preclinical* or pre-clinical*).tw. (124960)

146 or/143-145 (12204975)

147 142 and 146 (4183)

148 147 use emczd (2045)

149 72 or 148 (4172)

150 remove duplicates from 149 (3064)

***************************

**BIOSIS Previews**

2013 Jun 6

| # 30 | 988 | #29 AND #25  Databases=BIOSIS Previews Timespan=All years | |
| --- | --- | --- | --- |
| # 29 | 5,346,937 | | #28 OR #27 OR #26  Databases=BIOSIS Previews Timespan=All years |
| # 28 | 5,186,223 | | Taxa Notes=(Amphibians OR Artiodactyls OR Bats OR Birds OR Carnivores OR Cetaceans OR Dermopterans OR Edentates OR Elephants OR Fish OR Hyracoids OR Insectivores OR Lagomorphs OR Marsupials OR Monotremes OR Nonhuman Mammals OR Nonhuman Primates OR Nonhuman Vertebrates OR Pangolins OR Perissodactyls OR Pinnipeds OR Platyhelminths OR Pteridophytes OR Reptiles OR Rodents OR Sirenians) OR Taxonomic Data=(Pisces OR Amphibia OR Reptilia OR Aves OR Carnivora OR Cetacea OR Artiodactyla OR Chiroptera OR Dermoptera OR Edentata OR Hyracoidea OR Insectivora OR Lagomorpha OR Marsupialia OR Monotremata OR Perissodactyla OR Pinnipedia OR Pholidota OR Callithricidae OR Cebidae OR Cercopithecidae OR Daubentoniidae OR Indridae OR Lemuridae OR Lorisidae OR Pongidae OR Tarsiidae OR Tupaiidae OR Proboscidea OR Rodentia OR Sirenia OR Tubulidentata)  Databases=BIOSIS Previews Timespan=All years |
| # 27 | 2,299,504 | | Title=(animal$ or chordata or vertebrate* or fish or fishes or amphibian* or amphibium* or reptile$ or bird$ or mammal* or dog or dogs or canine$ or cat or cats or hyrax* or marsupial* or monotrem* or scandentia or bat or bats or carnivor* or cetacea or edentata* or elephant* or insect or insects or insectivore or lagomorph* or rodent* or mouse or mice or murine or murinae or muridae or rat or rats or pig or pigs or piglet$ or swine or rabbit$ or sheep$ or goat$ or horse$ or equus or cow or cows or cattle or calf or calves or bovine or sirenia or ungulate$ or primate$ or prosimian* or haplorhini* or tarsiiform* or simian*or platyrrhini or catarrhini or cercopithecidae or ape or apes or hylobatidae or hominid* or chimpanzee* or gorilla* or orangutan* or monkey or monkeys or ape or apes)  Databases=BIOSIS Previews Timespan=All years |
| # 26 | 41,445 | | Topic=(preclinical* or "pre-clinical" or "pre-clinically")  Databases=BIOSIS Previews Timespan=All years |
| # 25 | 1,792 | | #24 AND #15  Databases=BIOSIS Previews Timespan=All years |
| # 24 | 539,144 | | #23 OR #22 OR #21 OR #20 OR #19 OR #18 OR #17 OR #16  Databases=BIOSIS Previews Timespan=All years |
| # 23 | 31,663 | | Topic=((cecum or cecal or coecum or caecum) NEAR/3 (ligation* or puncture* or injur*)) OR Topic=(pancreatit*) OR Topic=(pancreas* NEAR/3 inflam*)  Databases=BIOSIS Previews Timespan=All years |
| # 22 | 116,340 | | Topic=(lipopolysaccharide* or "lipo-polysaccharide" or "lipo-polysaccharides" or LPS or lipoglycan*) OR Topic=("lung lavage") OR Topic=((bronchoalveolar or "broncho-alveolar" or alveolar or bronchial or lung$ or pulmonary or saline) NEAR/1 lavage*) OR Topic=((intravenous or IV or intrapulmonary or "intra-pulmonary" or intrabronchial or "intra-bronchial") NEAR/1 bacter*)  Databases=BIOSIS Previews Timespan=All years |
| # 21 | 71,199 | | Topic=("oleic acid") OR Topic=((oleic NEAR/1 acid*) or oleate or ("9-octadecenoic" NEAR/1 acid*) or ("cis-9-octadecenoic" NEAR/1 acid*)) OR Topic=(bleomycin* or blanoxan or blenoxane or "BLEO-cell" or bleolem or bleomicin*) OR Topic=(endotoxin* or ETX)  Databases=BIOSIS Previews Timespan=All years |
| # 20 | 15,928 | | Topic=(peritonitis) OR Topic=("aspiration pneumonia") OR Topic=((pneumonia or acid) NEAR/1 aspiration) OR Topic=(mendelson$ NEAR/1 syndrome)  Databases=BIOSIS Previews Timespan=All years |
| # 19 | 132,661 | | Topic=(sepsis or septic* or bacteremia or bacteraemia or endotoxic* or endotoxemi* or endotoxaemi*) OR Topic=(hyperoxi*) OR Topic=("reperfusion injury") OR Topic=((pulmonary or lung$ or nonpulmonary or non-pulmonary) NEAR/3 (ischemi* or ischaemi* or reperfusion*))  Databases=BIOSIS Previews Timespan=All years |
| # 18 | 29,066 | | Topic=("respiratory failure") OR Topic=((respiratory or ventilatory) NEAR/3 (insufficien* or deficien* or disturbance* or dysfunction* or depression or failure or paralys?s))  Databases=BIOSIS Previews Timespan=All years |
| # 17 | 8,643 | | Topic=(shock NEAR/1 lung) OR Topic=(acute NEAR/1 "lung injury") OR Topic=(acute NEAR/1 "lung injuries") OR Topic=(ALI)  Databases=BIOSIS Previews Timespan=All years |
| # 16 | 211,754 | | Topic=(lung$ NEAR/5 (patholog* or physiopatholog* or physio-patholog*)) OR Topic=((lung$ or pulmonary) NEAR/3 (disease* or injur* or contusion* or embolism* or haemorrhag* or hemorrhag* or infection* or trauma*)) OR Topic=("respiratory distress syndrome" AND (adult OR acute)) OR Topic=(ARDS or ARDSS)  Databases=BIOSIS Previews Timespan=All years |
| # 15 | 63,248 | | #14 OR #13 OR #12 OR #11 OR #10 OR #9 OR #8 OR #7 OR #6 OR #5 OR #4 OR #3 OR #2 OR #1  Databases=BIOSIS Previews Timespan=All years |
| # 14 | 2,320 | | Topic=(("Stem Cell Transplantation" OR "Gene Therapy") AND Mesenchymal)  Databases=BIOSIS Previews Timespan=All years |
| # 13 | 1,195 | | Topic=("IPS cell" or "IPS cells")  Databases=BIOSIS Previews Timespan=All years |
| # 12 | 34 | | Topic=("fibroblast-derived" NEAR/3 (stem or stroma$ or progenitor*) and (cell$ or "cell-induced"))  Databases=BIOSIS Previews Timespan=All years |
| # 11 | 9,033 | | Topic=(adult NEAR/3 (stem or stroma$ or progenitor*) and (cell$ or "cell-induced"))  Databases=BIOSIS Previews Timespan=All years |
| # 10 | 2,145 | | Topic=("adult stem cells")  Databases=BIOSIS Previews Timespan=All years |
| # 9 | 5,502 | | Topic=(mesoderm and cytolog*)  Databases=BIOSIS Previews Timespan=All years |
| # 8 | 911 | | Topic=("colony-forming unit" fibroblast* or "CFU-F" or "CFU-Fs")  Databases=BIOSIS Previews Timespan=All years |
| # 7 | 13,174 | | Topic=(MSC or MSCs or ADMSC or ADMSCs or BM-MSC or BM-MSCs or BMD-MSC or BMD-MSCs or BMDMSC or BMDMSCs or BMSC or BMSCs or HBMSC or HBMSCs or ABMSC or ABMSCs or MAPC or MAPCs)  Databases=BIOSIS Previews Timespan=All years |
| # 6 | 7,918 | | Topic=(("Bone Marrow Cells" or "Bone Marrow Transplantation") AND ("Stem Cells" or "Stem Cell Transplantation" or "Stem Cell Niche"))  Databases=BIOSIS Previews Timespan=All years |
| # 5 | 25,650 | | Topic=(marrow NEAR/3 (stem or stroma$ or progenitor*) and (cell$ or "cell-induced"))  Databases=BIOSIS Previews Timespan=All years |
| # 4 | 135 | | Topic=("multi-potent" NEAR/3 (stem or stroma$ or progenitor*) and (cell$ or "cell-induced"))  Databases=BIOSIS Previews Timespan=All years |
| # 3 | 4,167 | | Topic=(multipotent NEAR/3 (stem or stroma$ or progenitor*) and (cell$ or "cell-induced"))  Databases=BIOSIS Previews Timespan=All years |
| # 2 | 19,839 | | Topic=(mesenchymal NEAR/3 (stem or stroma$ or progenitor*) and (cell$ or "cell-induced"))  Databases=BIOSIS Previews Timespan=All years |
| # 1 | 15,566 | | Topic=("Mesenchymal Stem Cells") OR Topic=("Mesenchymal Stem Cell Transplantation") OR Topic=("Multipotent Stem Cells") OR Topic=("Mesenchymal Stromal Cells")  Databases=BIOSIS Previews Timespan=All years |

*988 hits*

**Web of Science**

*Science Citation Index Expanded (1900-present)*

*Conference Proceedings Citation Index- Science (1990-present)*

| # 29 | 1,125 | #28 AND #25  Databases=SCI-EXPANDED, CPCI-S Timespan=All years |
| --- | --- | --- |
| # 28 | 4,883,227 | #27 OR #26  Databases=SCI-EXPANDED, CPCI-S Timespan=All years |
| # 27 | 49,081 | Topic=(preclinical* or "pre-clinical" or "pre-clinically")  Databases=SCI-EXPANDED, CPCI-S Timespan=All years |
| # 26 | 4,858,177 | Topic=(animal$ or chordata or vertebrate* or fish or fishes or amphibian* or amphibium* or reptile$ or bird$ or mammal* or dog or dogs or canine$ or cat or cats or hyrax* or marsupial* or monotrem* or scandentia or bat or bats or carnivor* or cetacea or edentata* or elephant* or insect or insects or insectivore or lagomorph* or rodent* or mouse or mice or murine or murinae or muridae or rat or rats or pig or pigs or piglet$ or swine or rabbit$ or sheep$ or goat$ or horse$ or equus or cow or cows or cattle or calf or calves or bovine or sirenia or ungulate$ or primate$ or prosimian* or haplorhini* or tarsiiform* or simian*or platyrrhini or catarrhini or cercopithecidae or ape or apes or hylobatidae or hominid* or chimpanzee* or gorilla* or orangutan* or monkey or monkeys or ape or apes)  Databases=SCI-EXPANDED, CPCI-S Timespan=All years |
| # 25 | 2,524 | #24 AND #15  Databases=SCI-EXPANDED, CPCI-S Timespan=All years |
| # 24 | 551,851 | #23 OR #22 OR #21 OR #20 OR #19 OR #18 OR #17 OR #16  Databases=SCI-EXPANDED, CPCI-S Timespan=All years |
| # 23 | 44,107 | Topic=((cecum or cecal or coecum or caecum) NEAR/3 (ligation* or puncture* or injur*)) OR Topic=(pancreatit*) OR Topic=(pancreas* NEAR/3 inflam*)  Databases=SCI-EXPANDED, CPCI-S Timespan=All years |
| # 22 | 112,264 | Topic=(lipopolysaccharide* or "lipo-polysaccharide" or "lipo-polysaccharides" or LPS or lipoglycan*) OR Topic=("lung lavage") OR Topic=((bronchoalveolar or "broncho-alveolar" or alveolar or bronchial or lung$ or pulmonary or saline) NEAR/1 lavage*) OR Topic=((intravenous or IV or intrapulmonary or "intra-pulmonary" or intrabronchial or "intra-bronchial") NEAR/1 bacter*)  Databases=SCI-EXPANDED, CPCI-S Timespan=All years |
| # 21 | 81,243 | Topic=("oleic acid") OR Topic=((oleic NEAR/1 acid*) or oleate or ("9-octadecenoic" NEAR/1 acid*) or ("cis-9-octadecenoic" NEAR/1 acid*)) OR Topic=(bleomycin* or blanoxan or blenoxane or "BLEO-cell" or bleolem or bleomicin*) OR Topic=(endotoxin* or ETX)  Databases=SCI-EXPANDED, CPCI-S Timespan=All years |
| # 20 | 23,441 | Topic=(peritonitis) OR Topic=("aspiration pneumonia") OR Topic=((pneumonia or acid) NEAR/1 aspiration) OR Topic=(mendelson$ NEAR/1 syndrome)  Databases=SCI-EXPANDED, CPCI-S Timespan=All years |
| # 19 | 174,424 | Topic=(sepsis or septic* or bacteremia or bacteraemia or endotoxic* or endotoxemi* or endotoxaemi*) OR Topic=(hyperoxi*) OR Topic=("reperfusion injury") OR Topic=((pulmonary or lung$ or nonpulmonary or non-pulmonary) NEAR/3 (ischemi* or ischaemi* or reperfusion*))  Databases=SCI-EXPANDED, CPCI-S Timespan=All years |
| # 18 | 31,699 | Topic=("respiratory failure") OR Topic=((respiratory or ventilatory) NEAR/3 (insufficien* or deficien* or disturbance* or dysfunction* or depression or failure or paralys?s))  Databases=SCI-EXPANDED, CPCI-S Timespan=All years |
| # 17 | 17,188 | Topic=(shock NEAR/1 lung) OR Topic=(acute NEAR/1 "lung injury") OR Topic=(acute NEAR/1 "lung injuries") OR Topic=(ALI)  Databases=SCI-EXPANDED, CPCI-S Timespan=All years |
| # 16 | 163,849 | Topic=(lung$ NEAR/5 (patholog* or physiopatholog* or physio-patholog*)) OR Topic=((lung$ or pulmonary) NEAR/3 (disease* or injur* or contusion* or embolism* or haemorrhag* or hemorrhag* or infection* or trauma*)) OR Topic=("respiratory distress syndrome" AND (adult OR acute)) OR Topic=(ARDS or ARDSS)  Databases=SCI-EXPANDED, CPCI-S Timespan=All years |
| # 15 | 83,993 | #14 OR #13 OR #12 OR #11 OR #10 OR #9 OR #8 OR #7 OR #6 OR #5 OR #4 OR #3 OR #2 OR #1  Databases=SCI-EXPANDED, CPCI-S Timespan=All years |
| # 14 | 2,288 | TS=(("Stem Cell Transplantation" OR "Gene Therapy") AND Mesenchymal)  Databases=SCI-EXPANDED, CPCI-S Timespan=All years |
| # 13 | 1,520 | Topic=("IPS cell" or "IPS cells")  Databases=SCI-EXPANDED, CPCI-S Timespan=All years |
| # 12 | 23 | Topic=("fibroblast-derived" NEAR/3 (stem or stroma$ or progenitor*) and (cell$ or "cell-induced"))  Databases=SCI-EXPANDED, CPCI-S Timespan=All years |
| # 11 | 11,117 | Topic=(adult NEAR/3 (stem or stroma$ or progenitor*) and (cell$ or "cell-induced"))  Databases=SCI-EXPANDED, CPCI-S Timespan=All years |
| # 10 | 2,938 | TS=("Adult Stem Cells")  Databases=SCI-EXPANDED, CPCI-S Timespan=All years |
| # 9 | 15 | Topic=(mesoderm and cytolog*)  Databases=SCI-EXPANDED, CPCI-S Timespan=All years |
| # 8 | 638 | Topic=("colony-forming unit" fibroblast* or "CFU-F" or "CFU-Fs")  Databases=SCI-EXPANDED, CPCI-S Timespan=All years |
| # 7 | 17,595 | Topic=(MSC or MSCs or ADMSC or ADMSCs or BM-MSC or BM-MSCs or BMD-MSC or BMD-MSCs or BMDMSC or BMDMSCs or BMSC or BMSCs or HBMSC or HBMSCs or ABMSC or ABMSCs or MAPC or MAPCs)  Databases=SCI-EXPANDED, CPCI-S Timespan=All years |
| # 6 | 20,082 | TS=(("Bone Marrow Cells" or "Bone Marrow Transplantation") AND ("Stem Cells" or "Stem Cell Transplantation" or "Stem Cell Niche"))  Databases=SCI-EXPANDED, CPCI-S Timespan=All years |
| # 5 | 33,740 | Topic=(marrow NEAR/3 (stem or stroma$ or progenitor*) and (cell$ or "cell-induced"))  Databases=SCI-EXPANDED, CPCI-S Timespan=All years |
| # 4 | 101 | Topic=("multi-potent" NEAR/3 (stem or stroma$ or progenitor*) and (cell$ or "cell-induced"))  Databases=SCI-EXPANDED, CPCI-S Timespan=All years |
| # 3 | 4,127 | Topic=(multipotent NEAR/3 (stem or stroma$ or progenitor*) and (cell$ or "cell-induced"))  Databases=SCI-EXPANDED, CPCI-S Timespan=All years |
| # 2 | 29,088 | Topic=(mesenchymal NEAR/3 (stem or stroma$ or progenitor*) and (cell$ or "cell-induced"))  Databases=SCI-EXPANDED, CPCI-S Timespan=All years |
| # 1 | 25,915 | TS=("Mesenchymal Stem Cells") OR TS=("Mesenchymal Stem Cell Transplantation") OR TS=("Multipotent Stem Cells") OR TS=("Mesenchymal Stromal Cells")  Databases=SCI-EXPANDED, CPCI-S Timespan=All years |

*1125 hits*

**Web of Science**

#25 #24 AND #21

#24 #23 OR #22

#23 Topic=((preclinic* or pre-clinic*))

#22 Topic=((animal$ or chordata or vertebrate* or fish or fishes or amphibian* or amphibium* or reptile$ or bird$ or mammal* or dog or dogs or canine$ or cat or cats or hyrax* or marsupial* or monotrem* or scandentia or bat or bats or carnivor* or cetacea or edentata* or elephant* or insect or insects or insectivore or lagomorph* or rodent* or mouse or mice or murine or murinae or muridae or rat or rats or pig or pigs or piglet$ or swine or rabbit$ or sheep$ or goat$ or horse$ or equus or cow or cows or cattle or calf or calves or bovine or sirenia or ungulate$ or primate$ or prosimian* or haplorhini* or tarsiiform* or simian*or platyrrhini or catarrhini or cercopithecidae or ape or apes or hylobatidae or hominid* or chimpanzee* or gorilla* or orangutan* or monkey or monkeys or ape or apes))

#21 #20 AND #11

#20 #19 OR #18 OR #17 OR #16 OR #15 OR #14 OR #13 OR #12

#19 Topic=(((cecum or cecal or coecum or caecum) NEAR/3 (ligation* or puncture* or injur*)))

#18 Topic=(((bronchoalveolar or broncho-alveolar or alveolar or bronchial or lung$ or pulmonary or saline) NEAR/1 lavage*)) OR Topic=(((intravenous or IV or intrapulmonary or intra-pulmonary or intrabronchial or intra-bronchial) NEAR/1 bacter*))

#17 Topic=((oleic acid* or oleate or 9-octadecenoic acid* or cis-9-octadecenoic acid*)) OR Topic=((bleomycin* or blanoxan or blenoxane or BLEO-cell or bleolem or bleomicin*)) OR Topic=((endotoxin* or ETX)) OR Topic=((lipopolysaccharide* or lipo-polysaccharide* or LPS or lipoglycan*))

#16 Topic=(peritonitis) OR Topic=((pneumonia or acid) NEAR/1 aspiration) OR Topic=(hyperoxi*) OR Topic=(mendelson* NEAR/1 syndrome)

#15 Topic=(("inflammatory response syndrome" NEAR/1 systemic)) OR Topic=((sepsis or septic* or bacteremia or bacteraemia or endotoxic* or endotoxemi* or endotoxaemi*)) OR Topic=(hyperoxi*) OR Topic=(reperfusion NEAR/1 injur*) OR Topic=(((pulmonary or lung$ or nonpulmonary or non-pulmonary) NEAR/3 (ischemi* or ischaemi* or reperfusion*)))

#14 Topic=(ARDS or (shock NEAR/1 lung) or ALI) OR Topic=(((respiratory or ventilatory) NEAR/3 (insufficien* or deficien* or disturbance* or dysfunction* or depression or failure)))

#13 Topic=(respiratory distress syndrome AND (adult OR acute))

#12 Topic=((lung$ NEAR/5 (patholog* or physiopatholog* or physio-patholog* or disease* or injur*)))

#11 #10 OR #9

#10 Topic=(Stem Cell Transplantation OR Gene Therapy) AND Topic=(Mesenchymal)

#9 #8 OR #7 OR #6 OR #5 OR #4 OR #3 OR #2 OR #1

#8 Topic=(mesoderm and cytolog*)

#7 Topic=(colony-forming unit fibroblast* or CFU-F or CFU-Fs)

#6 Topic=((marrow stroma cell or marrow stromal cell or marrow stroma cells or marrow stromal cells))

#5 Topic=(MSC or MSCs or ADMSC or ADMSCs or BM-MSC or BM-MSCs or BMD-MSC or BMD-MSCs or BMDMSC or BMDMSCs)

#4 Topic=(multi-potent NEAR/3 (stroma$ or stem))

#3 Topic=(multipotent NEAR/3 (stroma$ or stem))

#2 Topic=((mesenchymal NEAR/3 (stem or stroma$ or progenitor*)))

#1 Topic=(Mesenchymal Stem Cells) OR Topic=(Mesenchymal Stem Cell Transplantation) OR Topic=(Multipotent Stem Cells) OR Topic=(Mesenchymal Stromal Cells)

*160 records*
